# Comparison of MRI and transthoracic echocardiography for detection of myocardial lesions in patients with coronary artery disease and preserved left ventricular function

**DOI:** 10.1186/1532-429X-15-S1-P207

**Published:** 2013-01-30

**Authors:** Philipp Halbfass, Marcel Mitlacher, Stefan Holzmann, Manfred Duecker, Konstantin Zintl, Johannes Brachmann, Christian Mahnkopf

**Affiliations:** 1 Dept. of Cardiology, Klinikum Coburg, Coburg, Germany

## Background

The presens of myocardial scar tissue as a potential arrhythmogen substrate may influence the treatment of patients with coronary artery disease (CAD). Delayed enhancement MRI (DE-MRI) allows accurate detection and visualization of myocardial scar tissue. We thought to detect myocardial scar tissue in patients with CAD and preserved left ventricular (LV) function without regional wall motion abnormalities (RWMA) in transthoracic echocardiography (TTE) using DE-MRI.

## Methods

83 patients (61 male; Age: 66.3±12.4 years) with a history of CAD and with normal LV function without RWMA in TTE were examined for viability determination using DE-MRI (MRI Verio 3T, Siemens, Erlangen, Germany).

## Results

**Figure 1:**
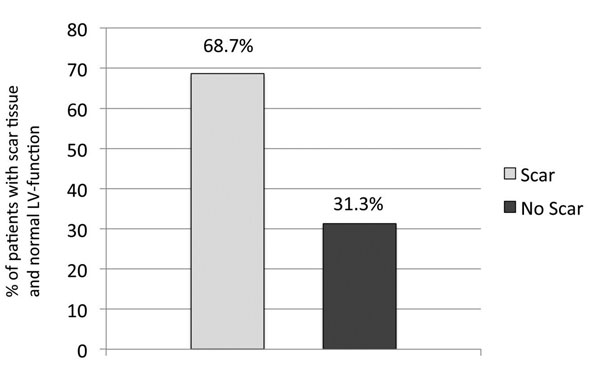
% of patients with scar tissue and normal LV-function

**Figure 2:**
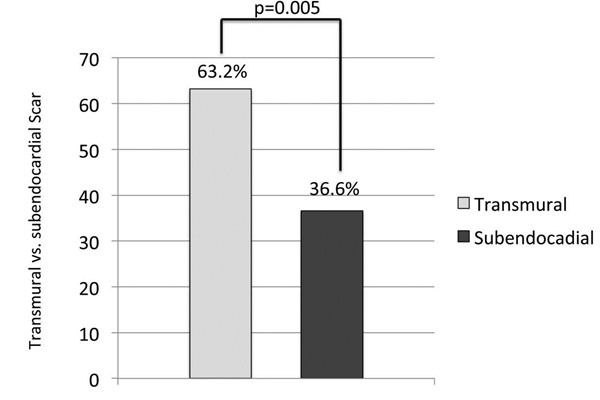
% of appearance of transmural and subendocardial scar tissue.

45 (54.2%) of these patients suffered from an acute coronary syndrome (elevated Troponin, NSTEMI, STEMI) shortly before the MRI examination while 8 patients were found with a history of ACS further in the past. Myocardial scar tissue was found in 57 patients (68.7%), whereas 26 patients (31.3%, Figure 1) showed no myocardial enhancement using DE-MRI. The number of patients with transmural scar was significantly higher than patients with subendocardial scar (36 (63.2%) vs. 21 (36.8%); p=0.005; Figure 2).

## Conclusions

From our preliminary results, a significant numbers of patients with CAD, preserved LV function and without regional wall motion abnormalities suffer from myocardial scar tissue. DE-MRI examinations of the heart for determination of myocardial viability should be considered in these patients to assess the extent of myocardial injury as TTE might underestimate the extent of myocardial scar tissue in patients with preserved left ventricular function.

## Funding

none

